# Revisiting the Temperature Dependence of the Photorespiratory CO_2_ Compensation Point (Γ*)

**DOI:** 10.1111/pce.70808

**Published:** 2026-08-12

**Authors:** Darwin L. Moreno‐Echeverry, Miko U. F. Kirschbaum, Margaret M. Barbour, Lìyǐn L. Liáng

**Affiliations:** ^1^ Manaaki Whenua Landcare Research Bioeconomy Science Institute Palmerston North New Zealand; ^2^ Te Aka Mātuatua – School of Science University of Waikato Hamilton New Zealand; ^3^ Institut de Recherche en Horticulture et Semences, INRAE University of Angers Beaucouzé France

**Keywords:** Arrhenius temperature function, C_3_ photosynthesis, CO_2_ release in the light, DL, FvCB model, gamma‐star, gas exchange, Laisk method, Rday, Rlight

## Abstract

Accurate estimation of the photorespiratory CO_2_ compensation point (Γ*) is essential for describing the balance between Rubisco carboxylation and oxygenation and for parameterising biochemical models of photosynthesis. Γ* and the rate of CO_2_ release in the light (*D*_L_) are commonly estimated using the Laisk method, based on measurements of net CO_2_ assimilation rate (*A*_net_) at low chloroplastic CO_2_ concentrations (c_c_), under several sub‐saturating irradiance levels. However, many widely used temperature dependence relationships for Γ* (Γ*(*T*)) were derived using conventional linear implementations of the Laisk method, despite the intrinsically nonlinear behaviour of the *A*_net_–c_c_ response predicted by the photosynthetic theory. Here, we revisited the temperature dependence of Γ* and *D*_L_ using the improved Laisk–FvCB framework that simultaneously constrains the nonlinear *A*_net_–c_c_ response across multiple irradiance levels. Gas exchange of sunflower leaves was measured across a wide temperature range from 3.9°C to 42.0°C. The conventional linear implementation generated highly dispersed pairwise intersections and unstable estimates of both Γ* and *D*_L_, including some physiologically unrealistic negative *D*_L_ values at low temperatures. In contrast, the mechanistically constrained Laisk–FvCB framework produced physiologically meaningful temperature responses and substantially reduced methodological artefacts associated with linear extrapolation. Using this framework, we derived a revised in vivo Γ*(*T*) relationship described by an Arrhenius‐type function with Γ*(25) = 43.4 μmol mol^−1^ and an apparent activation energy of 27.7 kJ mol^−1^, such that Γ*(*T*) = 43.4 exp[11.176 ((*T* − 25)/(*T* + 273.15))], where *T* is leaf temperature in °C. Comparison with other widely used Γ*(*T*) formulations showed substantial divergence at temperature extremes, often exceeding the variability expected from realistic interspecific differences in Rubisco specificity among C_3_ species.

## Summary statement

Using sunflower, we derived a revised in vivo temperature response of the photorespiratory CO_2_ compensation point (Γ*) based on a mechanistically constrained Laisk–FvCB framework. The analysis reveals that methodological assumptions can contribute substantially to discrepancies among published Γ*(*T*) functions.

## 1 Introduction

Quantifying the difference between CO_2_ assimilation and CO_2_ release is essential for understanding the carbon balance of C_3_ plant leaves. While plants assimilate CO_2_ through photosynthesis, a portion of the fixed carbon is released through two independent but connected processes: photorespiration and decarboxylation in the light (*D*_L_) (Dusenge et al. 2019; Moreno‐Echeverry, Kirschbaum, et al. 2026; Pärnik et al. 2007; Tcherkez et al. 2017). Photorespiration comes from the dual substrate specificity of Rubisco, which can bind both CO_2_ and O_2_ as substrates and catalyse either carboxylation or oxygenation of ribulose‐1,5‐bisphospate (RuBP) (Farquhar et al. 1980). The competition between carboxylation and oxygenation determines the photorespiratory CO_2_ compensation point (Γ*), defined as ‘the CO_2_ concentration at which CO_2_ uptake from carboxylation and CO_2_ loss from photorespiration are equal’ (Brooks and Farquhar 1985). Therefore, when the chloroplast CO_2_ concentration equals Γ*, the net assimilation rate (*A*_net_) equals *D*_L_.

As Γ* represents the CO_2_ compensation point in the absence of non‐photorespiratory respiration, it is determined by Rubisco specificity for CO_2_ relative to O_2_ (*S*_c/o_) and the O_2_ concentration at the site of Rubisco activity (Brooks and Farquhar 1985; Jordan and Ogren 1984; Walker et al. 2017; Zhang et al. 2024). Rubisco specificity for CO_2_ declines as temperature rises, and consequently oxygenation increases relative to carboxylation, resulting in an increase in Γ* (Galmés et al. 2016; Hermida‐Carrera et al. 2016; Jordan and Ogren 1984). Γ* is a fundamental parameter in the most widely used biochemical model developed by Farquhar, von Caemmerer, and Berry, also known as the FvCB model (Farquhar et al. 1980; Walker and Ort 2015; Yin, Busch, et al. 2020), so quantification of environmentally determined variation in the parameter is vital for accurate model predictions (Bernacchi et al. 2001).

Biochemical studies have quantified the temperature dependence of *S*_c/o_ in vitro and calculated the temperature dependence of Γ* [Γ*(*T*)] by combining the temperature dependence of Rubisco specificity with the relative change in solubility of CO_2_ and O_2_ (Jordan and Ogren 1984; Perdomo et al. 2015; Zhu et al. 1998). Alternatively, Γ*(*T*) can be determined in vivo using the Laisk method by measuring the CO_2_ evolution under different light levels at low CO_2_ concentrations (Laisk 1977; Brooks and Farquhar 1985).

The Laisk method has become one of the standard approaches for estimating Γ* and *D*_L_ from leaf gas exchange measurements, and its correct application is now widely discussed in methodological syntheses of photosynthetic measurements (Berghuijs et al. 2019; Busch et al. 2024; Villar et al. 1994; Walker and Cousins 2013). Although *D*_L_ has also been estimated using alternative approaches, including Kok‐type analyses (Gauthier et al. 2020; Way et al. 2019; Yin, Niu, et al. 2020) and combined gas exchange fluorescence techniques (Yin et al. 2011), the Laisk method remains widely used because it enables the simultaneous estimation of both Γ* and *D*_L_.

Estimates of Γ*(*T*) functions derived from enzyme assays of Rubisco and gas exchange measurements at the leaf level are broadly consistent (Jordan and Ogren 1984; Brooks and Farquhar 1985; Bernacchi et al. 2001), reflecting the relatively conserved nature of *S*_c/o_ among C_3_ plants (Galmés et al. 2016; Hermida‐Carrera et al. 2016). However, while being broadly consistent, notable discrepancies among published Γ*(*T*) relationships remain, particularly at low and high temperatures.

The importance of those discrepancies is illustrated in Figure 1, where we show an *A*_net_–c_c_ curve measured on a sunflower leaf at 20°C. The FvCB model was fitted to the data with Γ* set to different values based on three widely used literature sources, as indicated in the figure. Figure 1a shows the overall fit to the data, and Figure 1b shows an enlargement in the c_c_ range around Γ*. Similarly, good agreement between modelled and observed data could be obtained for the different assumed values of Γ*, but the choice of different Γ* values leads to different best parameter fits of other key parameters of the FvCB model, particularly for *D*_L_ (Figure 1b). The potential for parameter compensation within the FvCB framework becomes evident when Γ* is poorly constrained. The selected value of Γ* on the abscissa of the *A*_net_–c_c_ space, consequently, determines the value of *D*_L_ on the ordinate since *D*_L_ equals *A*_net_ at Γ*. Therefore, any error in estimating Γ* will lead to quantitatively important errors in *D*_L_ estimates (Gong et al. 2018; Moreno‐Echeverry, Kirschbaum, et al. 2026; Yin et al. 2011).

**Figure 1:**
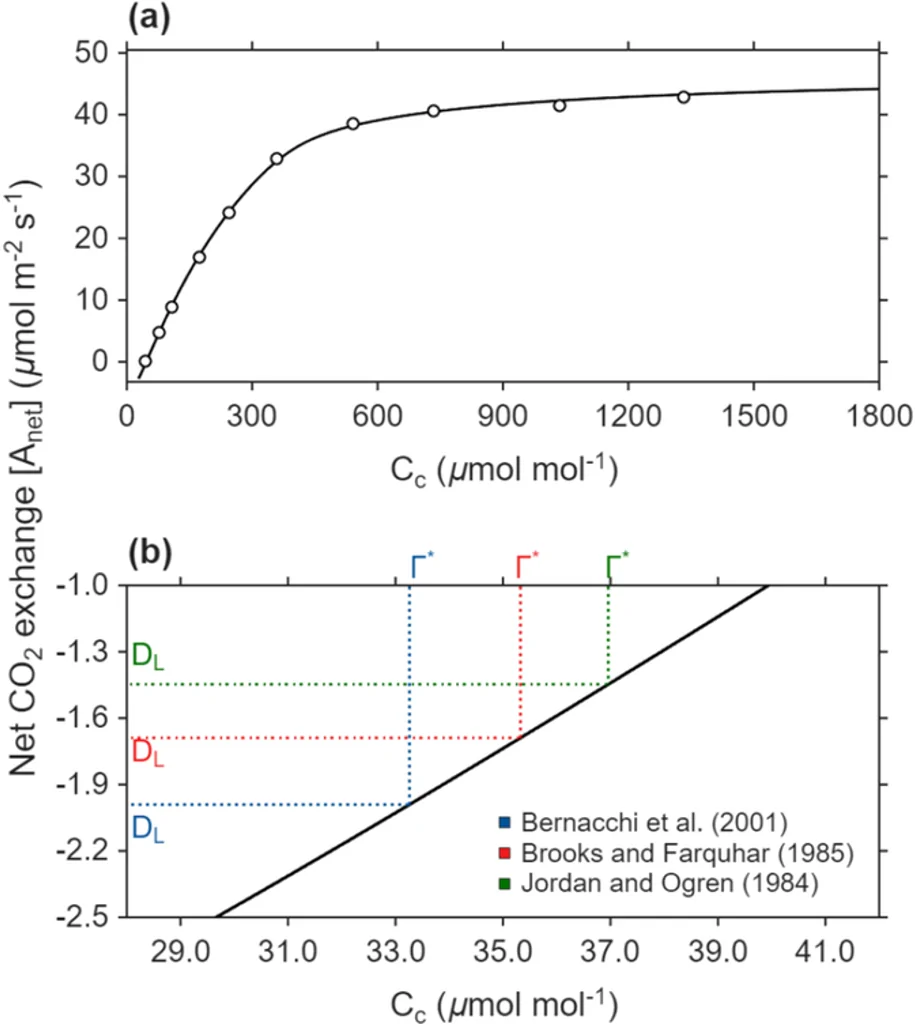
Schematic representation of the influence of Γ* on the estimation of *D*_L_ from an *A*_net_–c_c_ curve measured at 20°C. (a) The typical practice of fitting the FvCB model to the *A*_net_–c_c_ measurements on a leaf. (b) An enlargement of the low‐CO_2_ region showing how alternative Γ*(*T*) functions on the abscissa and lead to distinctly different *D*_L_ estimates for the same *A*_net_–c_c_ dataset. Vertical dotted lines indicate Γ* and horizontal dotted lines the corresponding *D*_L_.

In addition, previous estimates of Γ*(*T*) and *D*_L_(*T*) based on gas‐exchange measurements mainly relied on classical implementations of the Laisk method, in which linear regressions of *A*_net_ plotted against intercellular or chloroplast CO_2_ concentration were fitted over wide CO_2_ ranges. Previous work has shown that the nonlinear behaviour of the *A*_net_–c_c_ curve could have led to serious estimation errors (Moreno‐Echeverry, Kirschbaum, et al. 2026; Schmiege et al. 2023; Walker and Ort 2015). In most cases, these linear implementations may generate multiple pairwise intersections and unstable Γ* estimates across temperatures. Use of assumed linear relationships is likely to have introduced systematic bias in estimating Γ*, particularly at low and high temperatures, which will have propagated directly into estimates of *D*_L_. We have elaborated on these problems below.

In the present work, we have built on a recently developed integration of the Laisk method with the FvCB model that captures the nonlinearity of the *A*_net_–c_c_ curve (Moreno‐Echeverry, Kirschbaum, et al. 2026) and have used the modified approach to revisit the temperature dependence of Γ* and *D*_L_ using leaf gas exchange measurements. We compared conventional linear and nonlinear implementations of the Laisk method across a wide temperature range and evaluated whether methodological artefacts associated with linear fitting could contribute to discrepancies among published Γ*(*T*) relationships. We further assessed whether the variability among published Γ*(*T*) functions exceeded the range expected from interspecific variation in Rubisco specificity among C_3_ plants.

## 2 Materials and Methods

### 2.1 Plant Growth Conditions and Gas Exchange Measurements

Sunflower (*Helianthus annuus* ‘Russian Giant’) plants were grown in a plant growth unit. Plants were watered daily to field capacity and fertilised weekly with a complete nutrient solution. The photoperiod was 14/10 h, day/night, air temperatures were 24°C/19°C, and relative humidity was 40% during the day and 65% at night. Photosynthetic photon flux density (PPFD) at the leaf surface during the growth period was about 400 µmol photons m^−2^ s^−1^.

Sunflower was selected because it provides several practical and physiological advantages for temperature‐dependent gas exchange analyses. The species develops large, anatomically homogeneous leaves that facilitate stable IRGA measurements across broad temperature ranges while maintaining relatively high photosynthetic activity across short‐term thermal treatments and throughout the day.

Gas exchange was measured on fully expanded, upper canopy leaves using a LI‐6400XT portable photosynthesis system (LI‐COR Biosciences, Lincoln, NE, USA). Throughout the experiment, standard LI‐COR procedures were followed for flow rate control, IRGA matching, and H_2_O and CO_2_ calibration.

To enable measurements across a wide temperature range, the leaf cuvette of the LI‐6400XT was housed inside a purpose‐built temperature‐controlled enclosure. This insulated box allowed the ambient air surrounding the cuvette to be cooled or warmed independently of room temperature, ensuring stable and uniform thermal conditions during measurements. This configuration made it possible to achieve and maintain leaf temperatures as low as 3.9°C and as high as 42°C without condensation forming in the tubing or on optical surfaces. Air entering the system was equilibrated within the enclosure before reaching the analyser, which prevented humidity and temperature transients that could otherwise compromise IRGA stability at extreme temperatures.

Chamber air temperatures within the enclosure were adjusted over an approximate range of 5°C to 49°C, generating corresponding measured leaf temperatures ranging from 3.9°C to 42°C. All analyses were based on the measured leaf temperatures, which represent the physiologically relevant variable controlling photosynthetic kinetics. Differences between chamber air temperature and leaf temperature arose naturally from variation in leaf energy balance and transpirational cooling during measurements, particularly at temperature extremes. For each temperature response, leaves were acclimated for 20 min at the target leaf temperature, with chamber relative humidity maintained at approximately 60%, reference CO_2_ set to 400 μmol mol^−1^, and O_2_ maintained at the ambient atmospheric concentration of 21%. Within a given temperature treatment, successive *A*_net_–c_c_ curves were measured without returning leaves to 400 μmol mol^−1^ for a full reacclimation period between individual curves. Stable *A*_net_ values were confirmed before initiating each *A*_net_–c_c_ curve.

Because the application of the Laisk method requires three *A*_net_–c_c_ curves with distinct initial slopes, gas exchange measurements were conducted under three different sub‐saturating PPFD levels at each temperature. PPFD combinations were adjusted among leaves and temperature treatments to account for variation in leaf photosynthetic capacity and to ensure adequate separation among *A*_net_–c_c_ slopes under sub‐saturating conditions. *A*_net_–c_c_ curves were generated using stepwise decreases in reference CO_2_ concentration, ranging from approximately 200 down to 40 μmol mol^−1^, depending on temperature. Each Γ* and *D*_L_ estimate was therefore derived from a dataset consisting of three *A*_net_–c_c_ curves measured on the same leaf at a given temperature. In total, 56 Γ*– *D*_L_ datasets were obtained across the full range of temperature and irradiance conditions.

### 2.2 Determination of Γ* and *D*_L_ Based on Gas‐Exchange Measurements

At each target leaf temperature, Γ* and *D*_L_ were estimated using both the conventional Linear–Laisk method and a refined implementation of the Laisk method integrated with the FvCB photosynthesis model, as described by Moreno‐Echeverry, Avadie, et al. (2026). This implementation incorporates the smoothed transition between Rubisco‐limited and RuBP regeneration‐limited photosynthesis proposed by Kirschbaum and Farquhar (1984). In both approaches, three *A*_net_–c_c_ curves were measured over a low‐CO_2_ range under three sub‐saturating PPFD levels.

For the conventional Laisk method, linear regressions were fitted to the low CO_2_ region of each *A*_net_–c_c_ curve. Γ* and *D*_L_ were estimated from the mean of the pairwise intersections between regressions obtained under different levels within each dataset, where Γ* was taken as the *x*‐intercept and *D*_L_ as the *y*‐intercept. To assess the sensitivity of the conventional Laisk implementation to the arbitrary selection of the fitting interval, additional analyses were performed by fitting the linear regressions using the lowest three, four or five CO_2_ observations and comparing the resulting estimates with those obtained using the six‐point reference analysis. The corresponding results are presented in Figure S1.

In the nonlinear Laisk–FvCB method, values of Γ* and *D*_L_ were jointly estimated from the three curves and constrained to be identical across irradiance levels at a given temperature. Under these constraints, the fitted *A*_net_–c_c_ curves converged to a single common intersection in (c_c_, *A*_net_) coordinates, from which Γ* was defined as the *x*‐intercept and *D*_L_ as the *y*‐intercept. In both approaches, *D*_L_ was expressed as a positive flux corresponding to net CO_2_ release in the light. Model fitting simultaneously estimated the photosynthetic capacity and curvature parameters of the FvCB model (*V*_cmax_, *J*_max_, *α*, *θ* and *g*_m_), together with Γ* and *D*_L_. The optimisation procedure and parameter constraints are described in detail in Note S1.

### 2.3 Temperature Response of Γ* and *D*_L_

We applied the widely used Arrhenius function to describe the temperature dependence of Γ* and *D*_L_ as follows:

(1) $$X\left(T_{k}\right)=A\;\text{exp}\left(-\frac{E_{a}}{RT_{k}}\right),$$

where *X* denotes either Γ* or *D*_L_, *A* is the pre‐exponential factor, *E*_a_ is the activation energy (J mol^−1^), *R* is the universal gas constant (8.314 J mol^−1^ K^−1^), and *T*_k_ is absolute temperature in Kelvin.

This can be expressed in a normalised form relative to 25°C as

(2) $$X\;\left(T_{k}\right)=X\left(25\right)\;\text{exp}\left[\frac{E_{a}}{R}\;\left(\frac{1}{298.15}-\frac{1}{T_{k}}\right)\right],$$

where *X*(25) is Γ* or *D*_L_ at 25°C. Arrhenius functions were fitted in MATLAB R2026a using least squares regression to describe the temperature dependence of Γ* and *D*_L_.

For Γ*, we compared the resulting Γ*(*T*) relationship obtained using the Laisk–FvCB approach with the Arrhenius function reported by Bernacchi et al. (2001), parameterised with Γ*(25) = 42.8 μmol mol^−1^. In addition, we compared these Arrhenius functions with two polynomial descriptions reported by Brooks and Farquhar (1985): their own quadratic fit (Equation 3) and a second quadratic expression derived from the biochemical data of Jordan and Ogren (1984) (Equation 4):

(3) $$\Gamma^{*}\left(T\right)=42.7+1.68\left(T-25\right)+0.012{\left(T-25\right)}^{2},$$

(4) $$\Gamma^{*}\left(T\right)=44.7+1.88\left(T-25\right)+0.036{\left(T-25\right)}^{2},$$

where Γ* is in μmol mol^−1^ and *T* is leaf temperature in°C.

### 2.4 Statistical Comparison Between Methods

To evaluate whether the conventional Laisk–linear and Laisk–FvCB implementations produced significantly different parameter estimates, paired comparisons were performed using the 56 datasets analysed in this study. For each dataset, the difference between both implementations (Laisk–FvCB − Laisk–linear) was calculated for Γ* and *D*_L_. Normality of the paired differences was assessed using the Lilliefors test. When the paired differences followed a normal distribution, differences between methods were evaluated using a paired Student's *t*‐test; otherwise, a Wilcoxon signed‐rank test was used. To quantify the uncertainty of the estimated mean and median paired differences, bias‐corrected and accelerated (BCa) bootstrap confidence intervals were obtained from 10 000 paired bootstrap resamples. Effect sizes were calculated as Cohen's *d_z_*.

Agreement between both implementations was additionally assessed using Deming regression, which accounts for measurement uncertainty in both variables. Under perfect agreement, the regression intercept equals 0, and the slope equals 1 (*Y* = *X*). Bootstrap confidence intervals (10 000 paired resamples) were calculated for the intercept and slope, and a joint Wald test was used to evaluate the null hypothesis that the intercept equals 0 and the slope equals 1 simultaneously.

## 3 Results

### 3.1 Comparison of Linear and Nonlinear Implementations of the Laisk Method

The two implementations of the Laisk method produced fundamentally different representations of the *A*_net_–c_c_ relationship. The conventional Laisk–linear approach estimates Γ* and *D*_L_ from pairwise intersections among irradiance levels, whereas the Laisk–FvCB approach simultaneously constrains all irradiance levels using the nonlinear FvCB model (Figure 2). The differences between the two approaches were particularly evident at low temperatures. For example, at 4°C (Figure 2a), the pairwise linear regressions generated markedly different intersection points depending on the irradiance combination considered, whereas the Laisk–FvCB approach yielded a single physiologically consistent estimate.

**Figure 2:**
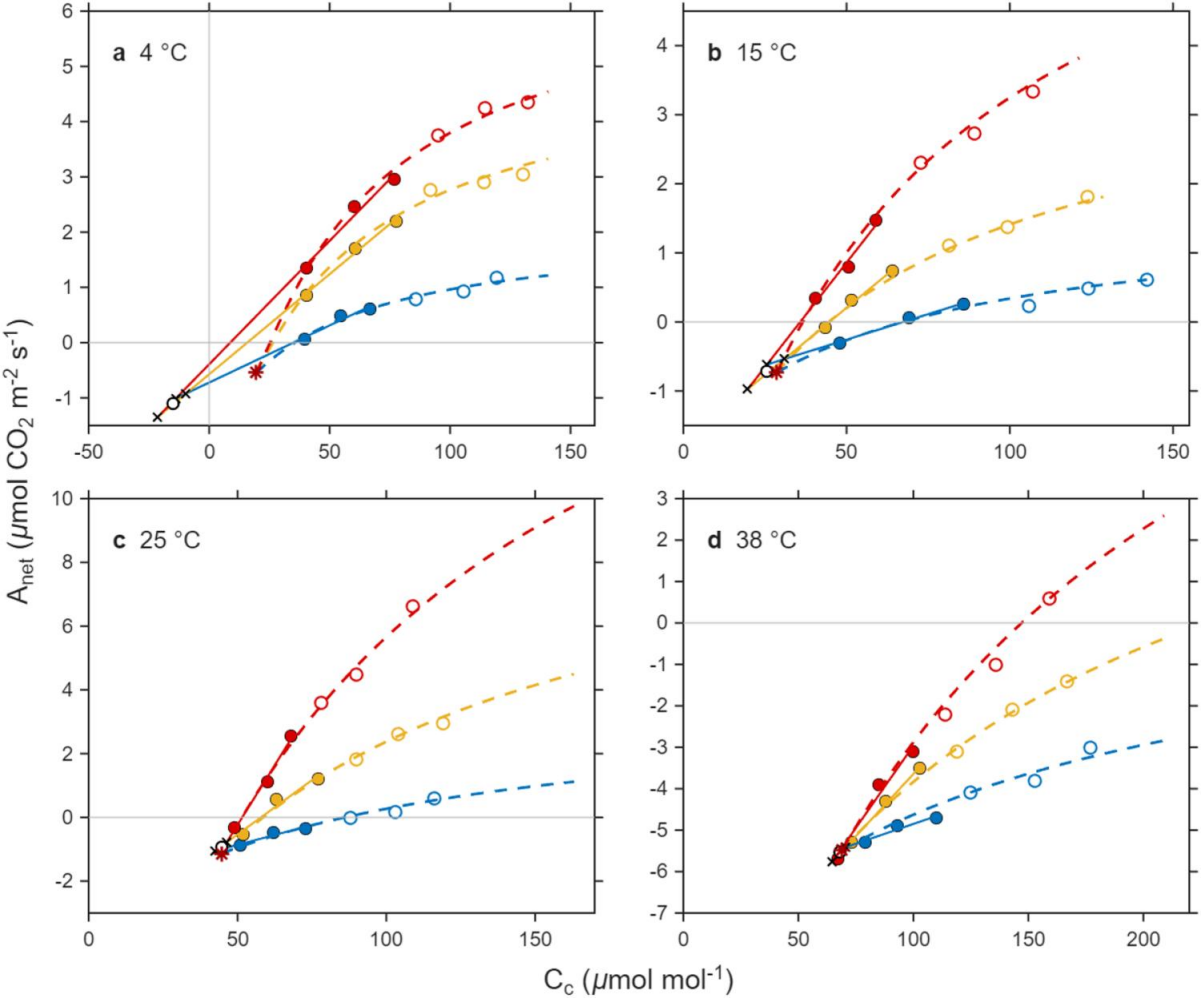
Representative *A*_net_–c_c_ curves at four leaf temperatures: (a) 4°C, (b) 15°C, (c) 25°C, and (d) 38°C. Coloured symbols represent measured net CO_2_ assimilation rates at three subsaturating light levels. Filled symbols indicate the three lowest c_c_ observations used to fit the conventional linear Laisk regressions, whereas open symbols indicate the remaining observations excluded from those linear fits. Solid coloured lines represent the corresponding linear regressions fitted to the intersections. Dashed coloured lines represent the nonlinear fits obtained with the Laisk–FvCB framework using the complete dataset at each irradiance level. Black crosses indicate the three pairwise intersections of the linear regressions, open black circles indicate their mean intersections, and red asterisks indicate the common intersection estimated with the Laisk–FvCB approach. [Color figure can be viewed at wileyonlinelibrary.com]

To further examine this variability, all individual pairwise intersections obtained with the conventional Laisk–linear approach were plotted across the full temperature range (Figure 3). Individual intersections showed substantial scatter, including highly divergent and physiologically unrealistic estimates of Γ*. Although averaging pairwise intersections reduced part of this variability, considerable dispersion remained across all temperatures, especially at low and high temperatures. In contrast, the Laisk–FvCB estimates followed a smooth and consistent trajectory throughout the entire temperature range.

**Figure 3:**
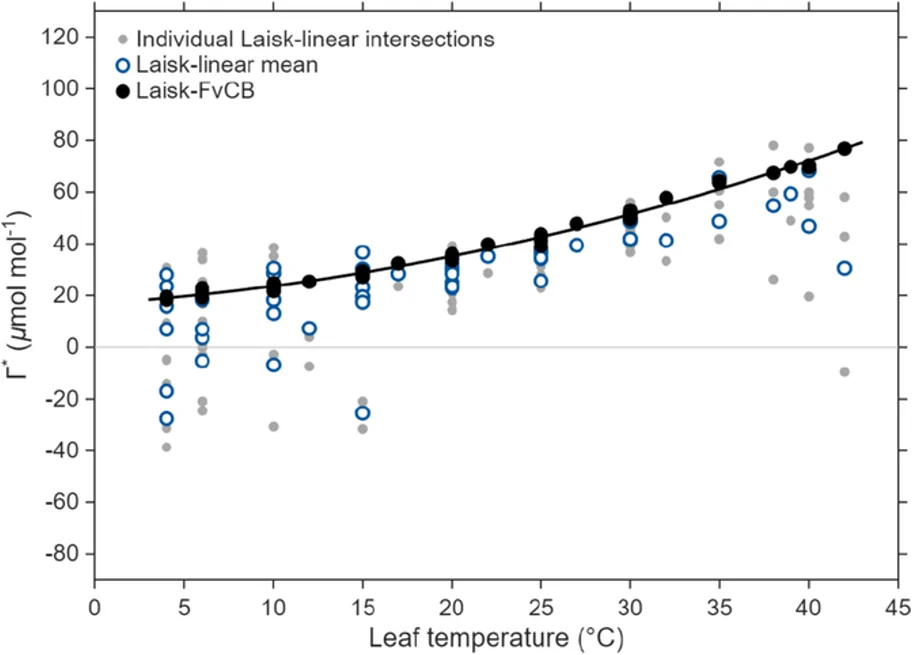
Comparison of temperature‐dependent Γ* (µmol mol^−1^) estimates obtained using the conventional Laisk–linear regression approach and the Laisk–FvCB method across multiple datasets and temperatures. Grey circles represent individual pairwise intersections obtained from linear regressions fitted to different light response combinations at each temperature. Open blue circles indicate the mean Γ* values calculated from the three pairwise Laisk–linear intersections for each temperature. Black circles represent Γ* estimates obtained using the Laisk–FvCB approach. [Color figure can be viewed at wileyonlinelibrary.com]

Although this discrepancy was most pronounced at low temperatures, the conventional implementation remained dependent on user‐defined linear regressions across all temperatures, whereas the Laisk–FvCB approach consistently provided a single mechanistically constrained estimate using the complete dataset. This dependence was further confirmed by repeating the linear analysis using different numbers of low‐CO_2_ observations. Estimates of both Γ* and *D*_L_ varied substantially with the selected fitting interval, whereas the nonlinear Laisk–FvCB approach was unaffected because it simultaneously fitted the complete dataset (Figure S1).

These methodological differences resulted in markedly different estimates of the temperature dependence of Γ* (Figure 4). The Laisk–FvCB approach generated a smooth and consistent increase in Γ* from approximately 20 μmol mol^−1^ at 3.9°C to 79 μmol mol^−1^ at 42°C (Figure 4a). In contrast, the conventional Laisk–linear approach produced substantially greater dispersion, particularly at lower temperatures, where several estimates approached 0 or became erroneously negative (Figure 4b). Although the mean Γ* values obtained with the linear approach also increased with temperature, the variability among datasets remained high across the entire temperature range.

**Figure 4:**
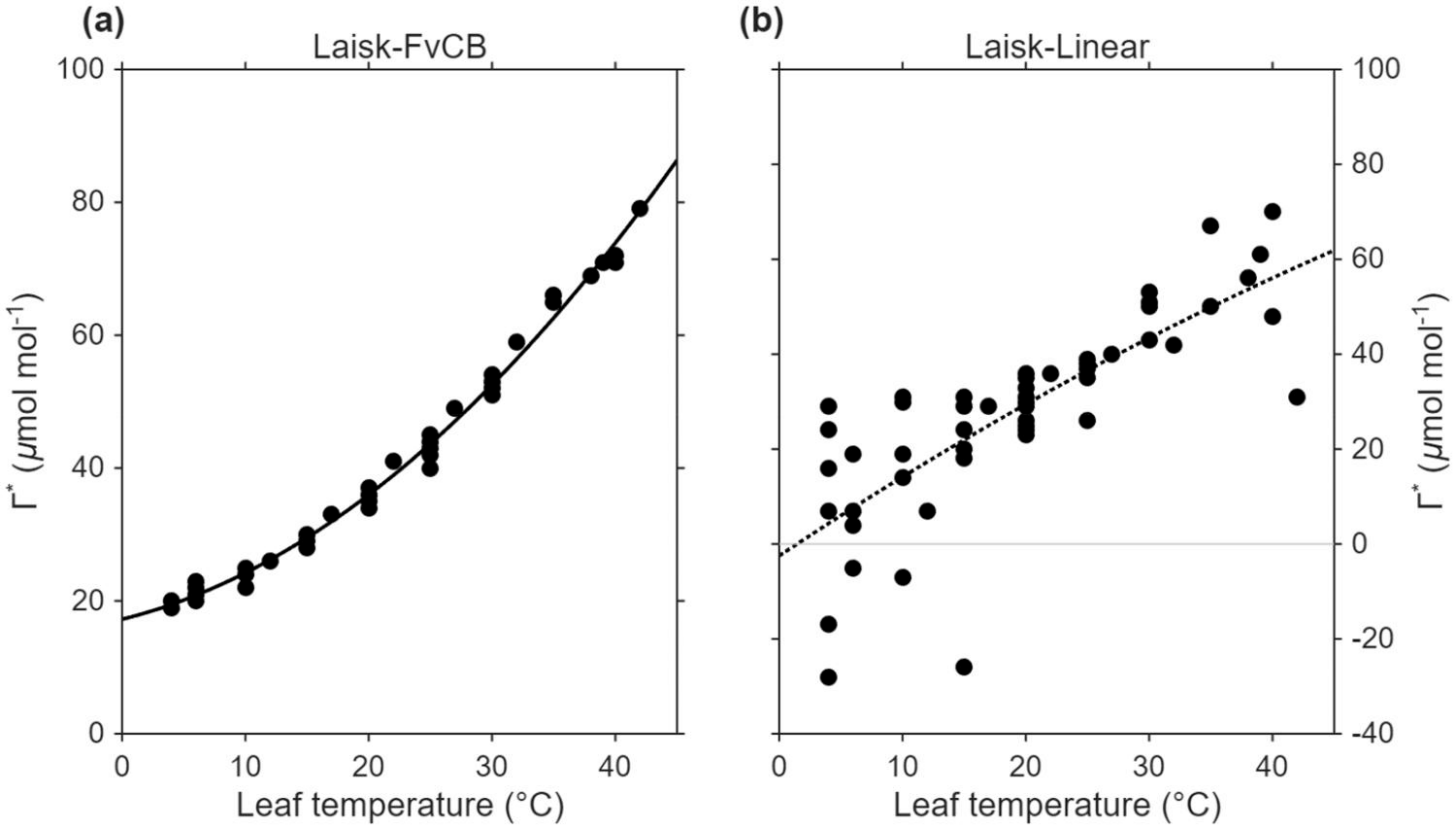
Temperature dependence of Γ* (µmol mol^−1^) for sunflower leaves. (a) Γ* estimated using the Laisk–FvCB approach as a function of leaf temperature. (b) Mean Γ* estimated using the conventional Laisk–linear regression approach. Each point represents the average of three pairwise intersections obtained from regressions fitted to the three light response datasets measured at each temperature.

### 3.2 Comparison of the Derived Temperature Dependence of *D*_L_

The contrasting behaviour observed for Γ* between the two implementations of the Laisk method also propagated into the estimation of *D*_L_ (Figure 5). The Laisk–FvCB approach produced a consistent increase in *D*_L_ with leaf temperature across the entire experimental range (Figure 5a). *D*_L_ remained positive at all temperatures and increased from 0.2 µmol CO_2_ m^−2^ s^−1^ at 3.9°C to 5 µmol CO_2_ m^−2^ s^−1^ at 42°C.

**Figure 5:**
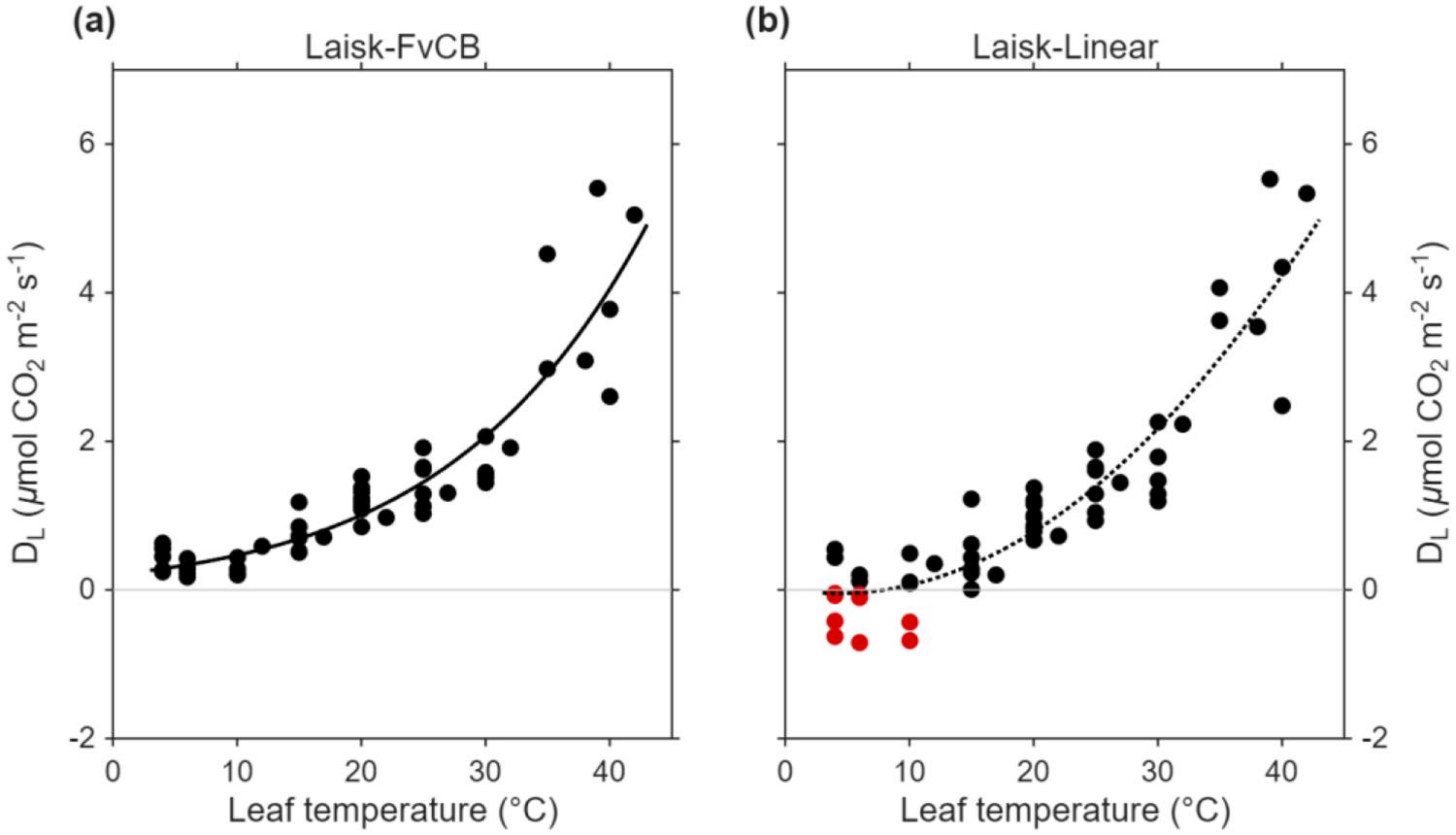
Temperature dependence of *D*_L_ estimated using the Laisk–FvCB and conventional Laisk–linear approaches for sunflower leaves. (a) *D*_L_ estimates obtained using the Laisk–FvCB method as a function of leaf temperature. (b) Mean *D*_L_ estimates obtained using the conventional Laisk–linear approach. Each point represents the average of the three pairwise intersections obtained from regressions fitted to the three irradiance datasets measured at each temperature. Red points indicate negative *D*_L_ values produced by the conventional Laisk–linear method. [Color figure can be viewed at wileyonlinelibrary.com]

In contrast, *D*_L_ estimates derived from the conventional Laisk–linear approach showed substantially greater variability (Figure 5b). Although the average *D*_L_ values generally increased with temperature, the dispersion among estimates remained high across the measured temperature range. Several *D*_L_ estimates became erroneously negative, particularly at low temperatures, reflecting the limitation of the conventional Laisk–linear method in accurately estimating *D*_L_. These negative values emerged directly from the inconsistent Γ* estimates generated by the multiple linear intersections observed in Figures 3 and 4.

These differences in *D*_L_ estimates were further confirmed when alternative published Γ*(*T*) were used during FvCB parameter estimation (Table S1). Whereas fitted *V*_cmax_ and *J*_max_ values remained comparatively stable across functions, *D*_L_ estimates showed substantial sensitivity to the selected Γ*(*T*) relationship, particularly at low and high temperatures.

### 3.3 Statistical Comparison of Parameter Estimates Obtained Using the Laisk–Linear and Laisk–FvCB Methods

To formally assess whether the two implementations produced significantly different parameter estimates, paired statistical comparisons were performed across the 56 gas exchange datasets analysed in this study (Table 1). The Laisk–FvCB implementation yielded systematically higher Γ* estimates than the conventional Laisk–linear implementation (Wilcoxon signed‐rank test, *p* = 1.95 × 10^−8^), with a mean paired difference of 9.91 µmol mol^−1^ (BCa bootstrap 95% confidence interval: 7.10–13.75 µmol mol^−1^). Likewise, *D*_L_ estimates were significantly higher using the Laisk–FvCB method (paired Student's *t*‐test, *p* = 2.32 × 10^−4^), with a mean paired difference of 0.180 µmol CO_2_ m^−2^ s^−1^ (BCa bootstrap 95% confidence interval: 0.091–0.268 µmol CO_2_ m^−2^ s^−1^).

**Table 1:** Paired statistical comparison of parameter estimates obtained using the Laisk–FvCB and conventional Laisk–linear implementations across the 56 gas‐exchange datasets analysed in this study.

Parameter	Mean paired difference (FvCB − linear)	BCa bootstrap 95% CI	Statistical test	*p* value	Cohen's *d_z_*
Γ* (µmol mol^−1^)	9.91	7.10–13.75	Wilcoxon signed‐rank	1.–95 × 10^−8^	0.79
*D*_L_ (µmol CO_2_ m^−2^ s^−1^)	0.180	0.091–0.268	Paired Student's (*t*)‐test	2.32 × 10^−4^	0.53

*Note:* Mean paired differences correspond to the difference between estimates obtained with the two implementations (Laisk–FvCB − Laisk–linear). Uncertainty was quantified using bias‐corrected and accelerated (BCa) bootstrap 95% confidence intervals based on 10 000 paired bootstrap resamples. Statistical significance was evaluated using either a Wilcoxon signed‐rank test or a paired Student's *t*‐test, depending on the distribution of the paired differences. Cohen's *d_z_* is reported as a measure of effect size.

Deming regression was used to assess the agreement between parameter estimates obtained with the two implementations (Figure 6). For Γ*, the fitted relationship was Γ*_FvCB_ = 16.54 + 0.757 Γ*_linear_. The intercept confidence interval excluded 0, indicating a significant constant disagreement between methods. Although the estimated slope was lower than unity, its bootstrap confidence interval included 1, indicating that proportional disagreement could not be established independently. Nevertheless, the joint hypothesis of zero intercept and unit slope was strongly rejected (*p* < 0.001).

**Figure 6:**
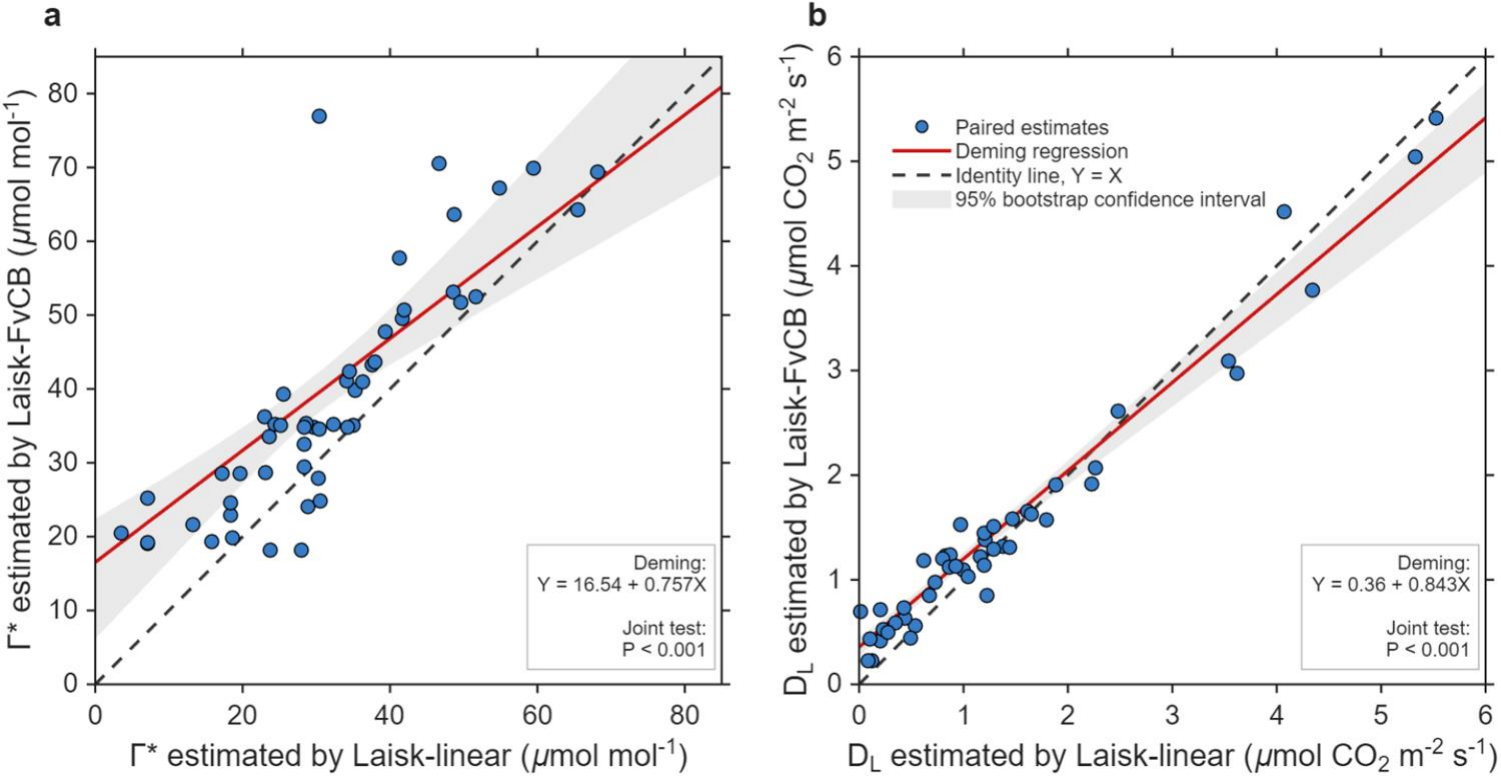
Agreement between the conventional Laisk–linear and Laisk–FvCB implementations for estimating Γ* and *D*_L_. (a) Comparison of Γ* estimates and (b) comparison of *D*_L_ estimates obtained from the 56 *A*_net_–c_c_ datasets analysed in this study. The dashed black line represents the line of identity (*Y* = *X*), indicating perfect agreement between methods. The solid red line shows the Deming regression, which accounts for measurement error in both variables. The grey shaded area represents the 95% bootstrap confidence interval of the fitted regression. Insets summarise the fitted regression equation and the *p*‐value of the joint test of the null hypothesis that the intercept equals 0 and the slope equals 1 (perfect agreement between methods). [Color figure can be viewed at wileyonlinelibrary.com]

For *D*_L_, the fitted relationship was *D*_L FvCB_ = 0.356 + 0.843 *D*_L linear_. The confidence intervals excluded both 0 for the intercept and unity for the slope, indicating both constant and proportional disagreement. Likewise, the joint hypothesis of perfect agreement was rejected (*p* < 0.001).

### 3.4 Comparison of Γ*(*T*) Functions and Temperature Sensitivity

The robust and internally consistent Γ* estimates obtained using the Laisk–FvCB approach allowed the derivation of an in vivo Γ*(*T*) relationship across the full experimental temperature range. Figure 7 compares the Γ*(*T*) derived in this study with three widely used formulations reported by Bernacchi et al. (2001), Brooks and Farquhar (1985), and Jordan and Ogren (1984).

**Figure 7:**
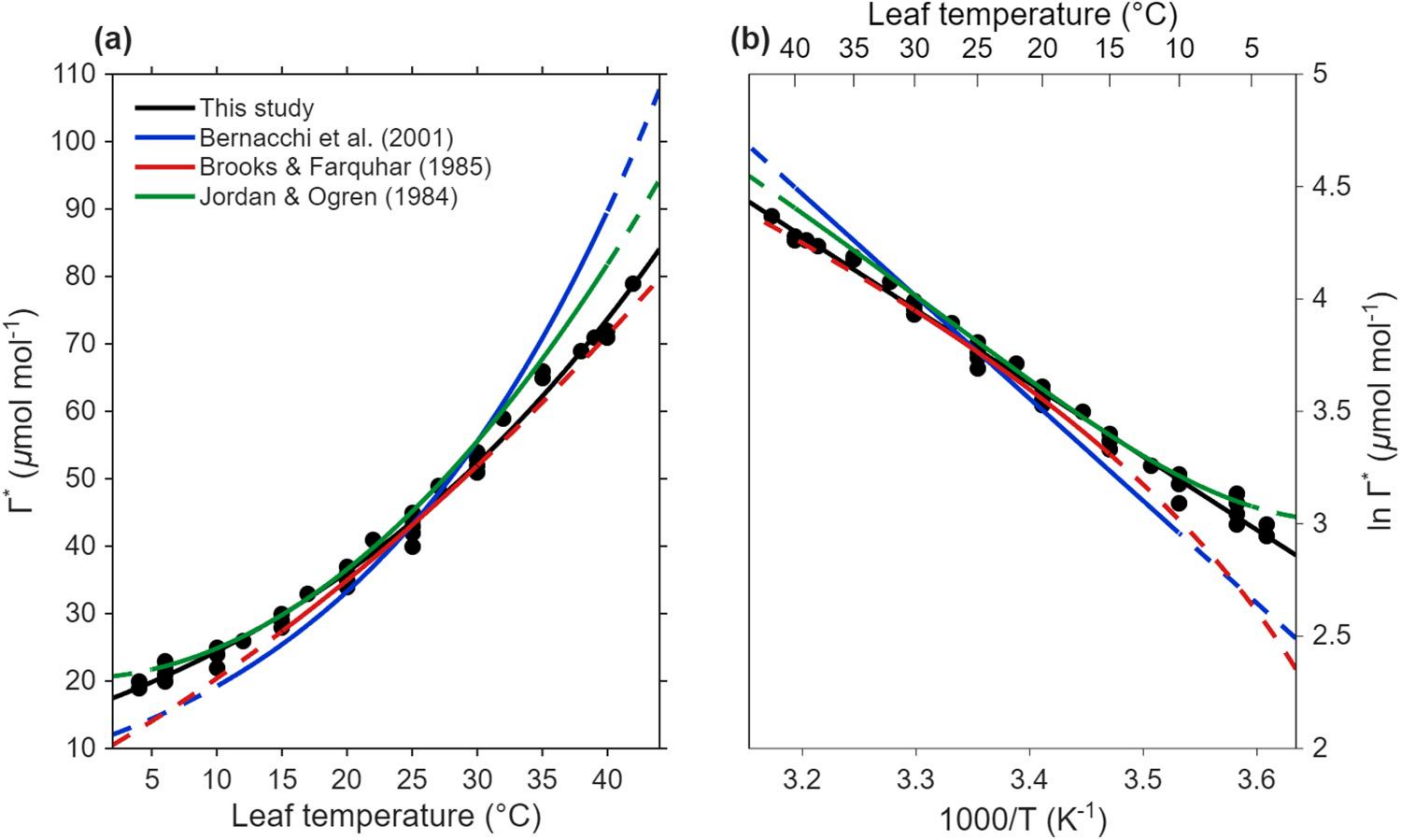
Comparison of alternative temperature dependence functions for Γ* (μmol mol^−1^). (a) Γ* as a function of leaf temperature, showing in vivo estimates from this study (solid black circles) together with the fitted Arrhenius and three commonly used Γ*(*T*) functions based on in vitro (Jordan and Ogren 1984) or gas‐exchange data (Brooks and Farquhar 1985; Bernacchi et al. 2001). (b) Same data and functions plotted in Arrhenius coordinates (ln Γ* vs. 1000/*T*), illustrating how alternative Γ*(*T*) functions differ in their implied temperature sensitivity. [Color figure can be viewed at wileyonlinelibrary.com]

At 25°C, we obtained a best‐fit Γ* of 43.4 μmol mol^−1^, compared with 42.8 μmol mol^−1^ from Bernacchi et al. (2001), 42.7 μmol mol^−1^ from Brooks and Farquhar (1985) and 44.7 μmol mol^−1^ from Jordan and Ogren (1984). We then examined how Γ* derived from the improved Laisk–FvCB fitting approach varied with leaf temperature. To provide a comparison with existing Γ*(*T*) functions from Jordan and Ogren (1984), Brooks and Farquhar (1985) and Bernacchi et al. (2001), we plotted the Γ*(*T*) function derived in the current study together with these functions (Figure 7a). Brooks and Farquhar (1985) reported Γ* values over the temperature range from 15°C to 30°C, whereas Jordan and Ogren (1984) and Bernacchi et al. (2001) covered wider temperature windows from 5°C to 40°C and from 10°C to 40°C, respectively.

To better compare the implied temperature sensitivities of these Γ*(*T*) functions, the same data and functions are shown in Arrhenius coordinates, with ln Γ* plotted against 1000/*T* (Figure 7b), showing clear differences in the slope of each function. Our Γ*(*T*) estimates closely followed a linear Arrhenius relationship over the full temperature range, yielding an apparent *E*_*a*_ of 27.7 kJ mol^−1^. The formulation of Bernacchi et al. (2001) produced a substantially steeper Arrhenius slope corresponding to a higher *E*_*a*_ of 37.8 kJ mol^−1^. By contrast, the polynomial functions of Brooks and Farquhar (1985) and Jordan and Ogren (1984) show marked curvature, especially at low temperatures, indicating departures from simple Arrhenius behaviour. This curvature reflects the limitations of applying polynomial Γ*(*T*) models outside their calibration domains and results in substantial divergence at the cold end of the range.

Although the alternative Γ*(*T*) functions diverged substantially from the relationship derived in this study, part of this variation could theoretically arise from interspecific differences in *S*_c/o_. To assess whether the observed deviation fell within the range expected from plausible biological variation among C_3_ species, we compared the relative differences among Γ*(*T*) functions with a reference envelope based on published variation in *S*_c/o_ (Figure 8).

**Figure 8:**
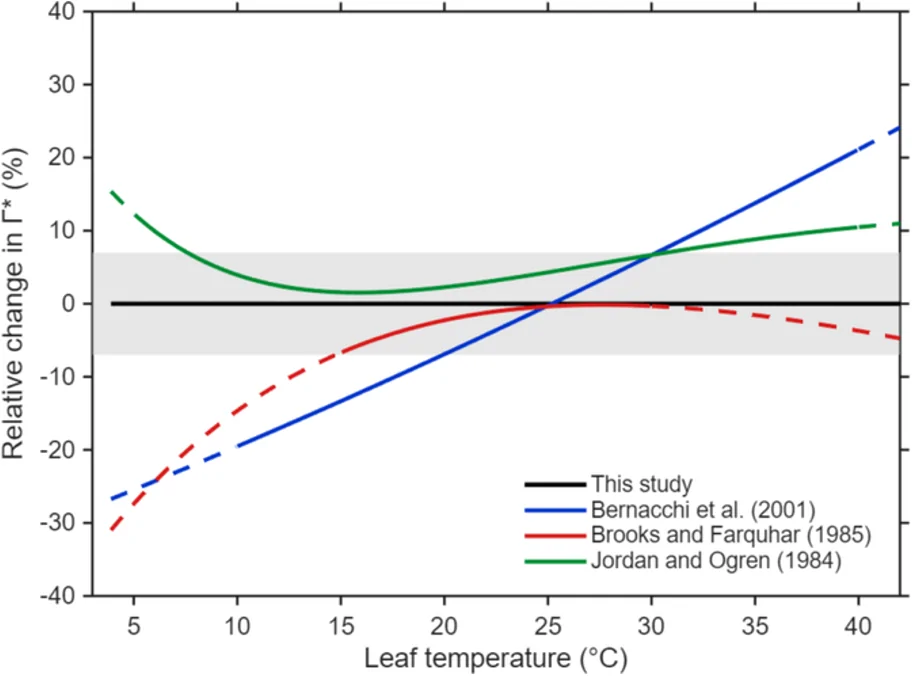
Relative differences in the temperature response of the photorespiratory CO_2_ compensation point (Γ*) between the Laisk–FvCB function and previously published Γ*(*T*) relationships. The black line represents the fitted reference Γ*(*T*) function obtained using the Laisk–FvCB framework in sunflower and therefore corresponds to zero relative deviation. Coloured lines show the relative deviation of the functions reported by Bernacchi et al. (2001) in blue, Brooks and Farquhar (1985) in red, and Jordan and Ogren (1984) in green from the reference function across the analysed temperature range. The grey shaded envelope represents the approximate ± 7% range of variation in Γ* expected from reported interspecific variability in *S*_c/o_ among C_3_ species. [Color figure can be viewed at wileyonlinelibrary.com]

The ±7% envelope represents a reference benchmark derived from the average interspecific variation in *S*_c/o_ compiled by Galmés et al. (2016). Their compilation included in vitro measurements of the temperature response of Rubisco specificity for 24 plant species spanning diverse C_3_ taxa and climatic origins (Table S2). This reference envelope is intended to provide an approximate indication of the magnitude of biologically realistic interspecific variation in *S*_c/o_, rather than to define the full range of variation observed among all species or genera. Indeed, larger differences have been reported for some taxonomic groups. For example, Orr et al. (2016) reported approximately 11% variation in *S*_c/o_ among *Oryza* species at 20°C, illustrating that the extent of natural variation can differ among taxa.

Because Γ* is inversely proportional to *S*_c/o_ (Γ* = 0.5 O/*S*_c/o_), a relative change in *S*_c/o_ propagates approximately one to one, but in the opposite direction, to Γ* when O_2_ concentration is assumed constant. Accordingly, a ± 7% change in *S*_c/o_ corresponds approximately to a ± 7% change in Γ*. Using the Γ*(*T*) function derived in this study as a reference, we constructed the corresponding ± 7% reference envelope and compared it with the relative differences produced by alternative Γ*(*T*) functions.

In general, the relative differences among Γ*(*T*) functions remained within the 7% reference envelope over moderate temperatures but increasingly exceeded this benchmark towards both low and high temperatures. The formulation of Bernacchi et al. (2001) showed the greatest divergence, reaching approximately ± 25% at the temperature extremes. The Brooks and Farquhar (1985) function reached 35% at the coolest temperature, and the Jordan and Ogren (1984) function also exceeded the expected range of variability, particularly at temperature extremes.

## 4 Discussion

### 4.1 Importance of a Methodologically Sound Basis for Reliable Γ*(*T*) Estimation

The Laisk method has become one of the most widely used gas exchange approaches for estimating Γ* and *D*_L_ (Yin and Amthor 2024; Zaragoza‐Castells et al. 2007). Estimates of Γ* are fundamental for parameterising photosynthetic models, and *D*_L_ plays a central role in understanding respiratory carbon losses and the coordination between photosynthesis and respiration under illuminated conditions (Farquhar et al. 1980; Tcherkez et al. 2017). Previous analyses have suggested that the conventional linear implementation of the Laisk method may introduce substantial methodological instability into the estimation of both Γ* and *D*_L_ (Walker and Ort 2015; Moreno‐Echeverry, Kirschbaum, et al. 2026).

The large dispersion observed among pairwise intersections demonstrates that this instability originates directly from the mathematical structure of the conventional linear implementation itself rather than from isolated experimental noise (Figures 2 and 3). Similar patterns emerged when the same methodological comparison was applied to the independent datasets of Way et al. (2019) and Crous et al. (2017). In both studies, like in the recent study (Figures 2 and 3), the conventional Laisk–linear implementation produced highly dispersed pairwise intersections across temperatures (Figures S2 and S3), which subsequently propagated into unstable and highly variable Γ* estimates (Figure S4). Together, these observations strongly indicate that the uncertainty is systematic and intrinsic to the linear approximation itself rather than dataset‐specific.

This uncertainty is mechanistically expected because the conventional Laisk implementation relies on linear regressions fitted over sections of the *A*_net_–c_c_ response, despite the intrinsically nonlinear behaviour predicted by the FvCB model (Farquhar et al. 1980). Under the traditional framework, the estimated intersection becomes highly sensitive to the selected CO_2_ range, irradiance level and measurement noise, particularly under low temperature conditions (Moreno‐Echeverry, Kirschbaum, et al. 2026), where photosynthetic rates decline and signal‐to‐noise ratios are inherently reduced. Consequently, part of the variability historically reported for Γ* (Walker and Ort 2015), likely reflects methodological uncertainty associated with the linear implementation rather than genuine physiological variation.

This dependence on the selected CO_2_ range introduces a fundamental trade‐off in the conventional implementation. Although restricting the fitting interval to observations closest to Γ* is theoretically desirable because this region is expected to be approximately linear, our sensitivity analysis showed that the estimated parameters remained highly sensitive to the selected fitting interval (Figure S1). Because the regression is fitted over a very narrow CO_2_ range, small changes in slope can produce large changes in the extrapolated intersection. Expanding the fitting interval improves slope constraint but progressively incorporates curvature into the fitted region, compromising the assumption of linearity. Consequently, no single choice of fitting interval consistently eliminates this source of uncertainty, as the conventional implementation must balance preserving local linearity against obtaining robustly constrained regressions.

The improved Laisk–FvCB framework substantially reduced these artefacts by embedding the Laisk logic directly within the mechanistic structure of the FvCB model and simultaneously constraining Γ* across multiple irradiance levels using the nonlinear *A*_net_–c_c_ response (Moreno‐Echeverry, Kirschbaum, et al. 2026).

### 4.2 Propagation of Γ* Uncertainty Into *D*_L_ Estimation

Accurate estimation of *D*_L_ is increasingly important for understanding daytime carbon balance because growing evidence indicates that CO_2_ release in the light is dynamic rather than constant and reflects the combined contributions of multiple metabolic processes (Tcherkez et al. 2017; Gong et al. 2018; Moreno‐Echeverry, Kirschbaum, et al. 2026). Despite these complexities and the continuing uncertainty surrounding its quantification, the Laisk framework remains one of the most widely used gas exchange approaches for estimating *D*_L_ in vivo.

The present results demonstrate that uncertainty in Γ* estimation propagates directly into estimates of *D*_L_. The conventional Laisk–linear implementation frequently generated highly variable *D*_L_ estimates and even produced negative values, particularly at low temperatures (Figure 5). Similar behaviour also emerged when the published datasets from Way et al. (2019) and Crous et al. (2017) were analysed using the same conventional implementation (Figure S5a), reinforcing the conclusion that these negative values most likely originate from methodological artefacts associated with unstable linear intersections. These observations strongly suggest that at least part of the physiologically unrealistic behaviour historically reported for *D*_L_ at low temperatures (Atkin et al. 2000; Tcherkez and Atkin 2021), may originate from uncertainty in Γ* estimation rather than from respiratory responses.

Under the improved Laisk–FvCB framework, *D*_L_ remained positive across the full temperature range examined and exhibited physiologically plausible thermal responses. This behaviour is consistent with current understanding of respiratory and photorespiratory carbon metabolism in illuminated leaves, where mitochondrial decarboxylation, photorespiration and carbon export processes interact dynamically with photosynthetic activity (Tcherkez et al. 2017; Gong et al. 2018).

### 4.3 Temperature Dependence of Γ*(*T*) and Its Implications for Photoynthesis Modelling

Temperature‐dependent Γ*(*T*) functions are widely used in photosynthesis models because Γ* quantifies the balance between Rubisco carboxylation and oxygenation within the FvCB framework. Many of the most commonly used Γ*(*T*) parameterisations originated either from implementations of the Laisk method (Brooks and Farquhar 1985; Bernacchi et al. 2001) or from biochemical approaches based on measurements of Rubisco specificity (Jordan and Ogren 1984). Consequently, uncertainties associated with the estimation methods propagate directly into subsequent modelling applications.

The Γ*(*T*) function derived here using the improved Laisk–FvCB framework exhibited a clear and monotonic increase with temperature across the measured range. This temperature response is broadly consistent with patterns reported in classical and more recent studies examining Rubisco specificity and photorespiratory kinetics (Brooks and Farquhar 1985; Galmés et al. 2016; Jordan and Ogren 1984; Perdomo et al. 2015; von Caemmerer et al. 1994).

In biochemical terms, Γ* increases with increases in the effective O_2_ concentration at the carboxylation site and decreases as *S*_c/o_ increases. Consequently, temperature influences Γ* through at least two coupled mechanisms: changes in the relative solubility of CO_2_ and O_2_, and changes in *S*_c/o_ itself. The increase in Γ* with warming, therefore, reflects well‐established biochemical and physical processes governing Rubisco kinetics, including the stronger temperature‐driven decline in CO_2_ solubility relative to O_2_, the increasingly favourable oxygenation transition state, and the progressive decline in *S*_c/o_ at higher temperatures (Bathellier et al. 2020; Cen and Sage 2005; Tcherkez et al. 2006; von Caemmerer et al. 1994). Together, these processes shift the balance of Rubisco reactions towards oxygenation at higher temperatures, resulting in a higher Γ*.

However, the Γ*(*T*) relationship derived using the Laisk–FvCB framework differed substantially from widely used published formulations, particularly at temperature extremes (Figure 6). Although part of this divergence could theoretically arise from genuine interspecific variation in *S*_c/o_ of the different species used in the studies, comparison with the variability compiled by Galmés et al. (2016) and Orr et al. (2016) demonstrated that the divergence among several published Γ*(*T*) functions exceeded the range expected solely from plausible biological variation among C_3_ species (Figure 7). These observations suggest that methodological differences among estimation frameworks likely contributed substantially to the variability among published Γ*(*T*) formulations. The Bernacchi et al. (2001) function showed the largest deviations, reaching approximately ± 25% relative to our reference relationship at the temperature extremes. At low temperatures, the Brooks and Farquhar (1985) function similarly diverged strongly from our reference relationship. These results do not demonstrate that any individual parameterisation is necessarily incorrect. Rather, they show that the divergence among widely used Γ*(*T*) functions can exceed the magnitude of variation typically reported for *S*_c/o_ among C_3_ species, particularly when the functions are applied outside the temperature ranges over which they were originally calibrated.

The contrasting behaviour of the two Laisk implementations further illustrates the magnitude of the methodological uncertainty introduced by the conventional linear approach. When individual Γ* estimates were expressed relative to the fitted Γ*(*T*) relationship derived from the Laisk–FvCB framework, their estimates remained tightly clustered around the reference relationship across the full temperature range (Figure S6a). In contrast, Γ* estimates obtained using the conventional Laisk–linear implementation showed extreme dispersion, with nearly all estimates falling outside the ± 7% variability range associated with realistic interspecific variation in *S*_c/o_ among C_3_ species (Figure S6b). Deviations commonly exceeded several tens of percent and reached values approaching 150% in some cases, demonstrating that the uncertainty introduced by the linear implementation greatly exceeds the magnitude of biologically plausible variation in *S*_c/o_.

These divergent trends become clearer in Arrhenius coordinates (Figure 6b), where the temperature response of the Γ*(*T*) function derived in this study was approximately linear across the measured temperature range. By contrast, the quadratic formulations displayed pronounced curvature towards the temperature extremes. This divergence became particularly evident outside the temperature ranges over which the original functions were calibrated, where extrapolation amplified differences among formulations. Consequently, although the quadratic functions may adequately describe Γ*(*T*) within their respective calibration domains, they produced increasingly divergent predictions at temperature extremes. Given the close relationship between Γ* and *S*_c/o_ (Jordan and Ogren 1984; Galmés et al. 2016; Perdomo et al. 2015), the approximately linear behaviour observed in Arrhenius coordinates suggests that an Arrhenius‐type formulation provides a robust empirical description of Γ*(*T*) across a broad thermal range.

Although the Arrhenius formulation of Bernacchi et al. (2001) also remained linear in Arrhenius space, it implied a substantially stronger temperature sensitivity than the relationship derived in the present study. Consequently, the Bernacchi formulation produced lower Γ* values at cool temperatures and higher values at warm temperatures, primarily because of its substantially higher apparent activation energy (*E*_a_ of 37.8 kJ mol^−1^) compared with the value estimated here (*E*_a_ of 27.7 kJ mol^−1^).

These methodological differences become particularly important when fitting FvCB models to conventional gas exchange datasets, where Γ*(*T*) is typically prescribed rather than estimated directly because the measurements do not contain sufficient information to constrain Γ* independently (Gu et al. 2010; Walker and Ort 2015). Under these conditions, the selected Γ*(*T*) relationship becomes a structural assumption of the model rather than an experimentally constrained variable. Consequently, any mismatch between the prescribed Γ*(*T*) function and the physiological Γ* consistent with the measured *A*_net_–c_c_ response must be compensated through adjustments in other fitted parameters.

The numerical analysis performed here demonstrated that alternative Γ*(*T*) formulations propagated strongly into fitted *D*_L_ estimates (Figure 1b), whereas *V*_cmax_ and *J*_max_ remained comparatively robust across the temperature range examined (Table S1). This behaviour is mechanistically expected because *D*_L_ acts as the principal compensatory parameter when Γ*(*T*) is imposed during fitting, whereas *V*_cmax_ and *J*_max_ are constrained more strongly by the broader structure of the *A*_net_–c_c_ response.

These findings have important implications for photosynthesis modelling because FvCB parameterisations commonly rely on previously published Γ*(*T*) functions rather than species‐specific estimates (Bernacchi et al. 2001; Sharkey et al. 2007; Walker and Ort 2015; Zaragoza‐Castells et al. 2007). The present results demonstrate that uncertainties associated with Γ*(*T*) estimation propagate into *D*_L_, thereby directly influencing the interpretation of daytime respiratory carbon fluxes and photorespiratory metabolism in model simulations.

## 5 Conclusion

We developed a revised Γ*(*T*) function based on in vivo gas exchange measurements spanning a wide temperature range from 3.9°C to 42.0°C. The resulting Γ*(*T*) function is described by an Arrhenius‐type relationship that can be expressed in normalised form as:

$$\Gamma^{*}\left(T\right)=43.4\;\text{exp}\left(11.176\;\frac{\left(T-25\right)}{\left(T+273.2\right)}\right).$$

This function was derived using the improved Laisk–FvCB framework, which provides a more mechanistically consistent representation of the nonlinear *A*_net_–c_c_ response across multiple irradiance levels, as demonstrated throughout the present study. Consequently, the revised Γ*(*T*) relationship reduces the methodological artefacts associated with conventional linear implementations of the Laisk method and provides a more robust basis for estimating photorespiration and daytime CO_2_ release across contrasting thermal conditions.

The improved framework also yielded a stable empirical temperature response of *D*_L_ across the measured temperature range, which can be described for the present *H. annuus* data set as:

$$D_{L}\left(T\right)=1.45\;\text{exp}\left(21.41\;\frac{\left(T-25\right)}{\left(T+273.2\right)}\right).$$

This relationship primarily characterises the shape of the temperature response observed under the experimental conditions of this study. The leaves used in this study had a mean nitrogen concentration of 2.82% on a dry‐mass basis, corresponding to approximately 0.92 g N m^−2^ based on a leaf mass per area of 32.52 g m^−2^. The absolute magnitude of *D*_L_, represented here by the reference value at 25°C, is expected to vary with species, leaf developmental and physiological status, growth conditions and, importantly, leaf nitrogen content. Thus, the temperature‐response function may serve as a useful basis for rescaling *D*_L_ to other plant material by adjusting its reference magnitude according to leaf nitrogen per unit area. Nevertheless, this relationship should not be interpreted as a universally validated parameterisation of daytime respiration.

## Supporting information


Supporting File

## Data Availability

The data that support the findings of this study are available in the supporting information of this article.
