# Supplementary material for: Revisiting the Temperature Dependence of the Photorespiratory CO2 Compensation Point (Γ*)
Source: Plant Cell Environ. 2026 Aug 12;49(11):9230–42. doi: 10.1111/pce.70808 (PMC13646666; doi:10.1111/pce.70808)
Supplement: Supplementary file 1 [file PCE-49-9230-s001.docx]

***Supplementary material***

Moreno-Echeverry, Darwin L., Kirshbaum, Miko U.F., Barbour, Margaret M. & Liáng, Lìyǐn L.

Revisiting the Temperature Dependence of the Photorespiratory CO_2_ Compensation Point (Γ*)

**Contents:**

**Supplementary table S1.** Uncertainty in Γ*(*T*) and its propagation to *D*_L_, *V*_cmax_ and *J*_max_.

**Supplementary table S2**. Temperature dependence of Rubisco specificity in C_3_ species from warm and cold environments.

**Supplementary Notes S1.** Iterative fitting procedure for Γ* and *D*_L_.

**Supplementary Notes S2.** The FvCB model of C_3_ photosynthesis.

**Supplementary Figure S1.** Sensitivity of parameter estimates obtained with the conventional linear Laisk implementation to the number of observations included in the linear regression.

**Supplementary Figure S2.** Comparison between the conventional Laisk-linear approach and the Laisk-FvCB method using gas exchange data from Way *et al*. 2019 measured in *Eucalyptus tereticornis* at four leaf temperatures.

**Supplementary Figure S3.** Comparison between the conventional Laisk-linear approach and the Laisk-FvCB method using gas exchange data from Crous *et al*. 2017 measured in *Eucalyptus globulus* at four leaf temperatures.

**Supplementary Figure S4.** Comparison of Γ* estimates obtained from the conventional Laisk-linear regression approach and the Laisk-FvCB method using published datasets from Way *et al*., 2019 and Crous *et al*., 2017.

**Supplementary Figure S5.** Comparison of *D*_L_ estimates obtained from the conventional Laisk-linear approach and the Laisk-FvCB method using published datasets from Way *et al*., 2019 and Crous *et al*., 2017.

**Supplementary Figure S6.** Relative change in the photorespiratory CO_2_ compensation point (Γ*) estimated using two different implementations of the Laisk method compared with the expected variation associated with the Rubisco specificity factor *S*_c/o_.

**Supplementary table S1.** Uncertainty in Γ*(T) and its propagation to *D*_L_, *V*_cmax_ and *J*_max_.

To quantify how uncertainty in Γ*(*T*) formulations influences estimates of *D*_L_ and the photosynthetic capacity parameters *V*_cmax_ and *J*_max_ when incorporated into the FvCB photosynthesis model, we performed a numerical analysis based on simulated *A*_net_–c_c_ curves. This approach allowed us to isolate the effects of alternative Γ*(*T*) formulations on parameter estimation, independent of experimental noise.

We simulated a collection of *A*_net_–c_c_ curves at four temperatures: 10, 20, 30 and 40 °C and then refitted the photosynthetic parameters under different prescribed Γ*(*T*). First, we generated a synthetic data set based on the Γ*(*T*) and *D*_L_(*T*) functions developed through the current work and set values of *V_c_*_max_ and *J*_max_ and their temperature dependence functions derived in previous work (unpublished data). We then changed Γ*(*T*) for each of the other three functions reported by Bernacchi *et al*. (2001), Brooks and Farquhar (1985) and Jordan and Ogren (1984) and refitted the FvCB model to the simulated data at each temperature to derive the best-fit *V*_cmax_, *J*_max_ and *D*_L_ parameters under each Γ*(*T*) applied. The simulated dataset gave us a consistent comparison by avoiding any effects of experimental errors on parameter estimations. The full set of FvCB equations in these fits is provided in the Supplementary Notes S2.

**TABLE S1.** Summary of fitted parameters from the FvCB model under the four Γ*(*T*) formulations at leaf temperatures of 10, 20, 30 and 40 °C. Columns report *D*_L_ (µmol CO_2_ m^-2^ s^-1^), *V*_cmax_ (µmol CO_2_ m^-2^ s^-1^), *J*_max_ (µmol electrons m^-2^ s^-1^), and the relative deviations (Δ*D*_L_, Δ*V*_cmax_, Δ*J*_max_; expressed in %) of each formulation with respect to our Γ*(*T*) reference function. Positive values indicate higher fitted values, and negative values indicate lower fitted values relative to this study. Model abbreviations: This study = reference parameterisation used here; **Be** = Bernacchi *et al.* (2001); **BF** = Brooks and Farquhar (1985); **JO** = Jordan and Ogren (1984).

| **Temp** | **Model** | ***D*_L_** | ***V*_cmax_** | ***J*_max_** | **Δ*D*_L_** | **Δ*V*_cmax_** | **Δ*J*_max_** |
| --- | --- | --- | --- | --- | --- | --- | --- |
| **10** | This study | 0.54 | 44.0 | 95.0 | - | - | - |
|  | Be | 0.65 | 44.0 | 95.2 | 90,3 | -1,4 | 0,3 |
|  | BF | 0.63 | 44.0 | 95.2 | 70,1 | -1,0 | 0,3 |
|  | JO | 0.51 | 44.0 | 94.9 | -25,0 | 0,3 | -0,1 |
|  |  |  |  |  |  |  |  |
| **20** | This study | 1.02 | 90.0 | 208.0 | - | - | - |
|  | Be | 1.13 | 90.1 | 208.2 | 38,4 | -0,1 | 0,1 |
|  | BF | 1.04 | 90.0 | 208.0 | 8,8 | 0,0 | 0,0 |
|  | JO | 0.98 | 90.0 | 207.9 | -14,7 | 0,0 | 0,0 |
|  |  |  |  |  |  |  |  |
| **30** | This study | 1.85 | 156.0 | 299.0 | - | - | - |
|  | Be | 1.67 | 155.9 | 298.7 | -26,8 | 0,1 | 0,1 |
|  | BF | 1.89 | 156.0 | 299.1 | 5,7 | 0,0 | 0,0 |
|  | JO | 1.69 | 155.9 | 298.8 | -23,7 | 0,0 | 0,1 |
|  |  |  |  |  |  |  |  |
| **40** | This study | 3.23 | 160.0 | 250.0 | - | - | - |
|  | Be | 2.66 | 159.7 | 249.4 | -35,9 | 0,1 | 1,0 |
|  | BF | 3.39 | 160.1 | 250.1 | 9,6 | 0,0 | -0,3 |
|  | JO | 2.94 | 159.9 | 249.7 | -18,2 | 0,1 | 0,5 |

Γ* estimates derived from the alternative formulations differed from our reference function by between -18.4 and 18.0 %, depending on temperature and model choice. These differences propagate strongly into fitted *D*_L_ estimates, which varied from -35.9 to +90.3 relative to the reference parameterisation. In contrast, fitted *V*_cmax_ and *J*_max_ remained comparatively stable, generally differing by less than 1.4 % across all Γ*(*T*) formulations and temperatures examined.

**Supplementary table S2**. Temperature dependence of Rubisco specificity in C_3_ species from warm and cold environments.

| Temperature (°C) | C_3_ warm (mol mol^-1^) | C_3_ cold (mol mol^-1^) | Average (mol mol^-1^) | Relative difference (%) |
| --- | --- | --- | --- | --- |
| 5 | 169,4 | 159,5 | 164,5 | 6,0 |
| 15 | 121,8 | 118,9 | 120,4 | 2,4 |
| 25 | 88,8 | 92,6 | 90,7 | -4,2 |
| 35 | 66,6 | 69,5 | 68,1 | -4,3 |
| 45 | 51,1 | 54,6 | 52,9 | -6,6 |

Values of the Rubisco specificity factor for CO_2_ relative to O_2_ (*S*_c/o_) for C_3_ species originating from warm (n = 16) and cool (n = 8) habitats, extracted from the compilation of Galmés *et al*. (2016), reported in Fig 1b. Values were used to quantify the magnitude of biologically plausible interspecific variation in Rubisco specificity. Relative differences were calculated as the difference between warm and cool habitat means divided by their average value at each temperature. Across the analysed temperature range, intergroup differences remained within approximately 2 to 7%, representing 24 C_3_ species spanning contrasting thermal environments. Because Γ* is inversely proportional to *S*_c/o_, these values were used to define the ±7% biological variation envelope shown in Figure 7.

**Supplementary Notes S1.** Iterative fitting procedure for Γ* and *D*_L_.

To improve numerical stability and ensure convergence of the common intersection across PPFD levels, parameter estimation was performed using a staged optimisation procedure for each leaf and temperature, using the full *A*_net_–c_c_ dataset from the three light intensities simultaneously. The same data were used at all stages, but different parameter constraints were applied to aid convergence.

**Stage 1**. All parameters were free (*V*_cmax_, *J*_max_, α, θ, *D*_L,_ Γ* and *g*_m_) to fit, using the combined dataset of n=18 points (3 light intensities × 6 c_c_ concentrations) for that leaf at each temperature.

**Stage 2**. Refit while holding *V*_cmax_, *J*_max_, α and θ fixed at their Stage 1 estimates and updating Γ*, *D*_L_ and *g*_m_, to stabilise the common intersection across PPFD levels.

**Stage 3**. final refit of Γ* and *D*_L_ with *g*_m_ held fixed.

All parameters were estimated with the Excel fitting template provided in Moreno-Echeverry *et al*. (2026), using Solver (GRG Nonlinear) to minimise the sum of squared residuals, within biologically realistic bounds.

**Supplementary Notes S2.** The FvCB model of C_3_ photosynthesis.

Net photosynthesis, *A*_net_, can be calculated in terms of the rate of Rubisco carboxylation, as

$$A_{net}=V_{c}\left( 1-\Gamma^{*}/c_{c} \right)-D_{L} (S1)$$

where *V*_c_ is the rate of carboxylation, Γ* is the photorespiratory CO_2_ compensation point, c_c_ is the chloroplast CO_2_ mole fraction and *D*_L_ is the rate of decarboxylation in the light. *V*_c_ can be determined as the minimum of three possible limiting factors: the Rubisco-limited carboxylation rate (*W*_c_), the carboxylation rate limited by RuBP (Ribulose 1,5-bisphosphate) regeneration or electron transport (*W*_j_), and the carboxylation rate constrained by phosphate availability (*W*_p_). Therefore, at a given chloroplastic CO_2_ partial pressure, *V*_c_ is modelled as the minimum of these three limiting rates (*W*_c_, *W*_j_, *W*_p_) as shown in Equation S2 (Kirschbaum & Farquhar, 1984).

$$V_{c} =\min\left\{ W_{c},W_{j},W_{p} \right\} (S2)$$

Since *W*_p_ was believed not to be limiting under our experimental conditions, this term was omitted here. *W*_c_ is then calculated as:

$$W_{c}=\frac{V_{cmax}^{'}\times c_{c}}{c_{c}+K_{m}} (S3)$$

where *V*_c_'_max_ is the apparent maximum carboxylation rate and *K*_m_ is the effective Michaelis-Menten constant for CO_2_ that also considers competitive inhibition by oxygen. In the calculation of *K*_m_, the partial pressure of oxygen (O_2_), the Michaelis-Menten constants for CO_2_ (*K*_c_) and for O_2_ (*K*_o_) are incorporated as seen in Equation 4.

$$K_{m} = K_{c} \left( 1+ \frac{O_{2}}{K_{o}} \right) (S4)$$

*V*´_cmax_ is calculated as:

$$V_{cmax}^{'} =\frac{V_{cmax}\times c_{c}}{c_{c}+K_{a}} (S5)$$

where *V*_cmax_ is the maximum carboxylation rate, and *K*_a_ is the Michaelis-Menten constant for activation of Rubisco by CO_2_ at a given Mg^2+^ concentration and pH (Kirschbaum & Farquhar, 1984). *W*_j_ is calculated as:

$$W_{j}=\frac{J*c_{c}}{4(c_{c}+2\Gamma^{*})} (S6)$$

where *J* is the potential electron transport rate and it is calculated as:

$$J= \frac{\left( \alpha*I+J_{max} \right)\pm\sqrt{\left( \alpha*I+J_{max} \right)^{2}-4\theta\left( \alpha*I*J_{max} \right)}}{2\theta} (S7)$$

where *J*_max_ is the maximum electron transport rate, α is the quantum yield of electron transport, I is the photon flux density of photosynthetically active radiation and θ describes the curvature of the light response of photosynthesis (Buckley & Farquhar, 2004). The transition between these limitations on *V*_c_ is not as abrupt as implied by Equation S2. The intrinsic nature of enzymatic kinetics and the variability in leaf tissue properties result in a region on the *A*_net_-c_c_ curve where different factors can co-limit the overall rates (Kirschbaum & Farquhar, 1984). Consequently, Equation S2 was rearranged by introducing an empirical factor (*e*), which transforms the function into a smooth hyperbola (Equation S8).

$$V_{c}^{2}-{V_{c} (W}_{c}+W_{j}+e)+W_{c}W_{j}=0 (S8)$$

By rearranging Equation 8, the transition between the limitation of the carboxylation rate due to both Rubisco and RuBP regeneration can be obtained, as shown in Equation 9.

$$V_{c}=\frac{\left( W_{c}+W_{j}+e \right) \pm\sqrt{\left( W_{c}+W_{j}+e \right)^{2}-4\left( W_{c}W_{j} \right)}}{2} (S9)$$

The resulting *V*_c_ from Equation 9 is then substituted into Equation 1. The estimated assimilation rate is thus calculated based on the FvCB model, incorporating modifications proposed by Kirschbaum and Farquhar (1984), and further refinements by Ubierna et al. (2019).

**Supplementary Figure S1.** Sensitivity of parameter estimates obtained with the conventional linear Laisk implementation to the number of observations included in the linear regression.

**FIGURE S1.** Sensitivity of parameter estimates obtained with the conventional linear Laisk implementation to the number of observations included in the linear regression. Differences in (a) Γ* and (b) *D*_L_ estimated using 3, 4 or 5 observations relative to estimates obtained using 6 observations (reference analysis) are shown for all 56 *A*_net_-c_c_ datasets. Each point represents an individual dataset. Boxplots show the median and interquartile range, with whiskers extending to 1.5 × the interquartile range. The dashed horizontal line indicates zero difference relative to the six-point analysis. Insets show the central distribution of the data, whereas the main panels retain all observations, including extreme values.

**Supplementary Figure S2.** Comparison between the conventional Laisk-linear approach and the Laisk-FvCB method using gas exchange data from Way *et al*. 2019 measured in *Eucalyptus tereticornis* at four leaf temperatures.

**FIGURE S2.** Comparison between the conventional Laisk-linear approach and the Laisk-FvCB method using gas exchange data from Way *et al.* 2019 measured in *Eucalyptus tereticornis* at four leaf temperatures (20, 25, 30 and 35°C). Panels show *A*_net_ as a function of c_c_ under three irradiance levels (300, 550, and 1800 μmol m^−2^ s^−1^). Solid coloured lines represent the linear regressions fitted over the full cc range originally reported by Way et al. (2019), including values extending beyond 100 μmol mol^−1^, whereas dashed coloured lines represent the fitted FvCB model simulations. Black asterisks indicate the individual pairwise intersections obtained from the linear regressions, open circles indicate the mean Laisk-linear intersection, and red asterisks indicate the common intersection estimated using the Laisk-FvCB approach. Insets magnify the intersection region in each panel to facilitate visual comparison between the two methods.

**Supplementary Figure S3.** Comparison between the conventional Laisk-linear approach and the Laisk-FvCB method using gas exchange data from Crous *et al*. 2017 measured in *Eucalyptus globulus* at four leaf temperatures.

**FIGURE S3.** Comparison between the conventional Laisk-linear approach and the Laisk-FvCB method using gas exchange data from Crous *et al*. 2017 measured in *Eucalyptus globulus* at four leaf temperatures (20, 28, 35 and 40°C). Panels show *A*_net_ as a function of c_c_ under three irradiance levels (200, 500, and 1800 μmol m^−2^ s^−1^). Solid coloured lines represent the linear regressions fitted over the full cc range originally reported by Crous *et al*. 2017, including values extending beyond 200 μmol mol^−1^, whereas dashed coloured lines represent the fitted FvCB model simulations. Black asterisks indicate the individual pairwise intersections obtained from the linear regressions, open circles indicate the mean Laisk-linear intersection, and red asterisks indicate the common intersection estimated using the Laisk-FvCB approach. Insets magnify the intersection region in each panel to facilitate visual comparison between the two methods.

**Supplementary Figure S4.** Comparison of Γ* estimates obtained from the conventional Laisk-linear regression approach and the Laisk-FvCB method using published datasets from Way *et al*., 2019 and Crous *et al*., 2017.

**FIGURE S4**. Comparison of Γ* estimates obtained from the conventional Laisk-linear regression approach and the Laisk-FvCB method using published datasets from Way *et al.*, 2019 (a) and Crous *et al*., 2017 (b). Grey circles represent the individual pairwise intersections obtained from linear regressions fitted to different light response combinations at each temperature. Open circles indicate the mean Γ* values calculated from the three pairwise Laisk-linear intersections for each temperature. Black circles represent Γ* estimates obtained using the Laisk-FvCB approach. Solid black curves represent quadratic fits to the Laisk-FvCB estimates.

**Supplementary Figure S5.** Comparison of *D*_L_ estimates obtained from the conventional Laisk-linear approach and the Laisk-FvCB method using published datasets from Way *et al*., 2019 and Crous *et al*., 2017.

**FIGURE S5.** Comparison of *D*_L_ estimates obtained from the conventional Laisk-linear approach and the Laisk-FvCB method using published datasets from Way *et al*., 2019 (a) and Crous *et al.*, 2017 (b). Grey circles represent the individual pairwise intersections obtained from linear regressions fitted to different light response combinations at each temperature. Open circles indicate the mean *D*_L_ values calculated from the three pairwise Laisk-linear intersections for each temperature. Black circles represent *D*_L_ estimates obtained using the Laisk-FvCB approach. Solid black curves represent quadratic fits to the Laisk-FvCB estimates. Red outlined circles indicate negative *D*_L_ estimates produced by the conventional Laisk-linear approach.

**Supplementary Figure S6.** Relative change in the photorespiratory CO_2_ compensation point (Γ*) estimated using two different implementations of the Laisk method compared with the expected variation associated with the Rubisco specificity factor *S*_c/o_.

**FIGURE S6.** Relative change in the photorespiratory CO_2_ compensation point (Γ*) estimated using two different implementations of the Laisk method. Panel (a) shows the relative deviation of Γ* estimates obtained using the Laisk–FvCB framework from the fitted reference Γ*(*T*) curve derived from the same dataset. Panel (b) shows the corresponding relative deviation obtained using the conventional linear Laisk approach. The grey shaded envelope represents a conservative ±7 % range of expected variation in Γ* associated with interspecific variability in Rubisco specificity factor (*S*_c/o_) among C_3_ species, derived from the temperature responses reported by Galmés *et al*. (2016). Open circles represent individual Γ* estimates at each measurement temperature.
